# Developing a risk prediction model for the functional outcome after hip arthroscopy

**DOI:** 10.1186/s12891-018-2030-x

**Published:** 2018-04-19

**Authors:** Patrick Stephan, Maarten A. Röling, Nina M. C. Mathijssen, Gerjon Hannink, Rolf M. Bloem

**Affiliations:** 1 Orthopaedic Research Laboratory, Radboud University Medical Center, Nijmegen, The Netherlands; 2 Department of Orthopaedics, Reinier de Graaf Groep, Delft, The Netherlands

**Keywords:** Hip arthroscopy, Risk prediction, Clinical prediction rule, Functional outcome, Hip outcome score, Preoperative decision-making

## Abstract

**Background:**

Hip arthroscopic treatment is not equally beneficial for every patient undergoing this procedure. Therefore, the purpose of this study was to develop a clinical prediction model for functional outcome after surgery based on preoperative factors.

**Methods:**

Prospective data was collected on a cohort of 205 patients having undergone hip arthroscopy between 2011 and 2015. Demographic and clinical variables and patient reported outcome (PRO) scores were collected, and considered as potential predictors. Successful outcome was defined as either a Hip Outcome Score (HOS)-ADL score of over 80% or improvement of 23%, defined by the minimal clinical important difference, 1 year after surgery. The prediction model was developed using backward logistic regression*.* Regression coefficients were converted into an easy to use prediction rule.

**Results:**

The analysis included 203 patients, of which 74% had a successful outcome. Female gender (OR: 0.37 (95% CI 0.17–0.83); *p* = 0.02), pincer impingement (OR: 0.47 (95% CI 0.21–1.09); *p* = 0.08), labral tear (OR: 0.46 (95% CI 0.20–1.06); *p* = 0.07), HOS-ADL score (IQR OR: 2.01 (95% CI 0.99–4.08); *p* = 0.05), WHOQOL physical (IQR OR: 0.43 (95% CI 0.22–0.87); *p* = 0.02) and WHOQOL psychological (IQR OR: 2.40 (95% CI 1.38–4.18); p = < 0.01) were factors in the final prediction model of successful functional outcome 1 year after hip arthroscopy. The model’s discriminating accuracy turned out to be fair, as 71% (95% CI: 64–80%) of the patients were classified correctly.

**Conclusions:**

The developed prediction model can predict the functional outcome of patients that are considered for a hip arthroscopic intervention, containing six easy accessible preoperative risk factors. The model can be further improved trough external validation and/or adding additional potential predictors.

## Background

Arthroscopic intervention in the hip joint has evolved as a successful therapeutic procedure over the last decades for treating various causes of hip complaints. As diagnostic skills and surgical techniques continue to improve in managing these hip disorders, the indications for hip arthroscopy are also expanding [1, 2]. Hip arthroscopy is primarily used in the treatment of femoroacetabular impingement (FAI) caused by cam and/or pincer morphology, labral tears, focal articular cartilage injuries or the removal of loose bodies in the joint [3]. Treatment of these conditions can lead to pain relief, improvement of hip function [2] and might delay the onset of osteoarthritis and the progression to total hip arthroplasty (THA) [4, 5].

Despite that an arthroscopic treatment for FAI in general is successful, not all patients equally benefit from this procedure [6]. As with any operative procedure, multiple studies emphasize the importance of proper patient selection in achieving favorable results [7–12]. Unsuccessful treatment, e.g. insufficient reduction of complaints, requiring revision surgery or even short term progression to total hip arthroplasty caused by progressive osteoarthritis, has been associated with different preoperative factors. Progressive osteoarthritis has proven to have a negative effect on outcome results of patients treated for FAI [7–13]. A high Tönnis classification (grade ≥2) and a reduced joint space (< 2 mm) have been described as exclusion criteria for hip arthroscopy [7]. Literature suggests that age, gender, BMI, duration of symptoms, preoperative outcome scores, preoperative alpha-angle and hip dysplasia could be predictive for the outcome after hip arthroscopic surgery [7–11, 13–17].

A clinical prediction model would be a great asset in making it easier to predict the outcome of individual patients that are considered for a hip arthroscopic intervention, and could be used to guide doctors and patients in shared decision making regarding treatment and expectations [18, 19]. Therefore, the purpose of this study was to develop a clinical prediction model that can be used to predict the functional outcome 1 year after hip arthroscopy.

## Methods

### Study population

This study is a retrospective analysis of routinely collected data on all patients who underwent a hip arthroscopic intervention in our hospital between April 2011 and March 2015. All data were collected prospectively. Two hundred five consecutive patients underwent a hip arthroscopic intervention and surgery was performed by an experienced single orthopedic surgeon (RMB). Inclusion criteria for hip arthroscopy were a cam and/or a pincer deformity or a suspicion of a labral tear. The diagnosis was made based on clinical examination with a combination of complaints (hip/groin pain or functional disability), physical examination (FADIR and FABER tests as described by Phillippon et al. [20]) and the presence of radiographic findings that correlate with FAI hip pathology. Patients with severe signs of hip osteoarthritis (Tönnis grade 3) were not offered hip arthroscopy and were therefore excluded from the study. Also, patients unwilling to participate were excluded. All included patients were asked to fill out patient reported outcome (PRO) questionnaires preoperative and at postoperative follow up at 3 months and 1 year. Patient assessment did not differ from normal clinical practice.

The questionnaires used to assess improvement in patient outcome were the Visual Analogue Scale (VAS) for pain, modified Harris Hip Score (mHHS), Hip Outcome Score (HOS)-ADL and HOS-Sport and the physical and psychological domains of the WHOQOL. The mHHS, HOS-ADL and HOS-Sport were used to asses hip related improvement in patient outcome, which are scored percentage based on 8, 17 and 9 questions respectively. The WHOQOL-BREF [21] score was used to measure the general (non hip related) quality of life (QOL). The WHOQOL score is a generic measure designed for use in a wide spectrum of psychological and physical disorders. It is a multidimensional measure for subjective assessment of QOL. The WHOQOL-BREF has a good to excellent validity and reliability [22]. High scores indicate a good QOL. Patients in the study were scored on both the physical and psychological domains. The study protocol was assessed by the regional Medical Ethical Committee (Medisch Ethische Toetsings Commissie Zuidwest Holland (METCZH); no. METCZWH 12–083). Ethical approval was waived by the METCZH on basis of the Dutch Medical Research Involving Human Subjects Act (WMO). However, all the patients who were included gave their written informed consent. Our study was reported according to the TRIPOD guidelines [23].

### Surgical technique

Patients were operated in supine position under general anesthesia. A traction table was used for subluxation of the hip joint. Fluoroscopy guided, two to three portals were inserted into the hip joint in order to adequately visualize the acetabulum, acetabular labrum, cartilage, transverse ligament and the anterior, superior and posterior aspects of the femoral head. The central and peripheral compartments were inspected for abnormalities (as described by Bond 2009) [24]. Labral tears, focal chondropathy, loose bodies, cam- and/or pincer morphologies were identified and treated accordingly: tears were repaired if possible, otherwise debrided, cam/pincer morphologies resected, loose bodies extracted and focal chondropathy > grade II were treated with microfracture.

### Outcome measure

To be able to predict the risk of a successful outcome it was required to define a cutoff in the HOS-ADL, which is used as the main outcome score. To do this the Minimal Clinically Important Difference (MCID) for the HOS-ADL was used. The MCID is a common tool used to determine the smallest change in a treatment outcome that a patient would identify as important. In a recent study Chahal et al. reported an MCID of 23 for the HOS-ADL [25]. Also patients scoring above 80% in HOS-ADL score were classified as having an successful outcome [14].

Ultimately, a successful outcome was defined as either a 23% improvement in HOS-ADL from preoperative to 1 year postoperative, or a HOS-ADL score of over 80% at 1 year postoperative.

### Potential predictive factors

Based on literature [7–17], the following potential predictors were considered: age, gender, years of complaints, BMI, operation indication (cam, pincer, labral tear), preoperative radiographic findings (Tönnis classification, alpha angle) and PRO scores (VAS for pain, mHHS, HOS-ADL, HOS-Sport, WHOQOL physical and psychological domains). The predictors were either continuous (age, years of complaints, BMI, alpha angle, and all outcome scores), dichotomous (gender) or categorical (indication, Tönnis classification) and used as such.

### Statistical analysis

Descriptive statistics were used to summarize the data. For 12 of the 21 variables, data were missing ranging between 1% to 18% (Table 1). These missing data were imputed using multiple imputation by chained equations procedure (predictive mean matching) [26, 27]. Missing data were assumed to be missing at random (MAR), five imputed datasets were created.

**Table 1:** Demographic and clinical characteristics of study participants

Characteristic	Total sample (*n* = 203)		Patients with successful outcome^a^ (*n* = 133)		Patients with unsuccessful outcome^a^ (*n* = 46)		Patients with missing outcome (*n* = 24)	
	Value	Missing	Value	Missing	Value	Missing	Value	Missing
Gender (female)	114 (56%)	–	64 (48%)	–	33 (72%)	–	17 (71%)	–
Age (yrs)	40 (11, 15–67)	–	40 (11, 17–67)	–	40 (10, 18–55)	–	39 (13, 15–64)	–
BMI (kg/m^2^)	26 (4, 18–42)	–	25 (4, 18–35)	–	26 (5, 20–42)	–	27 (6, 20–40)	–
Duration of complaints (yrs)	4 (4, 1–30)	–	4 (4, 1–25)	–	3 (2, 1–10)	–	5 (6, 1–30)	–
Tönnis classification	193	10 (5%)	128	5 (4%)	45	1 (2%)	20	4 (17%)
Grade 0	142 (74%)	–	94 (73%)	–	35 (78%)	–	13 (65%)	–
Grade 1	46 (23%)	–	32 (25%)	–	9 (20%)	–	5 (25%)	–
Grade 2	5 (3%)	–	2 (2%)	–	1 (2%)	–	2 (10%)	–
Alpha angle (^o^)	64 (14, 39–99)	–	66 (14, 39–99)	–	62 (13, 39–91)	–	62 (14, 43–89)	–
Cam	120 (59%)	–	87 (65%)	–	20 (44%)	–	13 (54%)	–
Pincer	45 (22%)	–	26 (20%)	–	12 (26%)	–	7 (29%)	–
Labral tear	138 (68%)	–	87 (65%)	–	37 (80%)	–	14 (58%)	–
Preoperative PRO scores								
VAS pain, mean	6 (2, 1–10)	–	6 (2, 1–10)	–	6 (2, 2–9)	–	7 (1,3–9)	–
mHHS, mean (SD, range)	55 (12, 22–90)	–	57 (12, 26–90)	–	53 (11, 23–80)	–	50 (14, 22–72)	–
HOS-ADL, mean (SD, range)	58 (20, 7–96)	2 (1%)	60 (20, 7–96)	–	53 (18, 14–84)	–	55 (22, 15–91)	2 (8%)
HOS-Sport, mean (SD, range)	40 (22, 0–94)	11 (5%)	42 (23, 0–94)	3 (2%)	38 (19, 3–78)	3 (7%)	34 (22, 0–72)	5 (21%)
WHOQOL physical, mean (SD, range)	49 (16, 7–86)	30 (15%)	50 (16, 7–86)	17 (13%)	48 (14, 21–82)	6 (13%)	45 (19, 7–75)	7 (29%)
WHOQOL psychological, mean (SD, range)	71 (15, 13–100)	29 (14%)	72 (15, 13–100)	16 (12%)	67 (16, 25–95)	6 (13%)	69 (16, 38–96)	7 (29%)
PRO scores at 3 months postoperative								
VAS pain	3 (2, 0–9)	12 (6%)	2 (2, 0–9)	6 (5%)	4 (3, 0–8)	1 (2%)	2 (2, 0–6)	5 (21%)
mHHS	73 (15, 17–91)	12 (6%)	77 (12, 41–91)	5 (4%)	63 (18, 17–91)	1 (2%)	69 (15, 41–87)	6 (25%)
HOS-ADL	76 (20, 27–100)	118 (58%)	81 (15, 38–100)	77 (58%)	64 (23, 28–100)	27 (59%)	73 (25, 27–100)	14 (58%)
HOS-Sport	62 (27, 6–100)	129 (64%)	65 (24, 6–100)	83 (62%)	51 (32, 6–100)	29 (63%)	63 (26, 33–97)	17 (71%)
WHOQOL physical								
WHOQOL psychological								
PRO scores at 1 year postoperative								
VAS pain	3 (3, 0–9)	20 (10%)	1 (2, 0–9)	–	5 (2, 0–9)	–	6 (4, 1–9)	20 (83%)
mHHS	77 (16, 27–91)	25 (12%)	84 (9, 47–91)	1 (1%)	57 (15, 27–91)	3 (7%)	60 (31, 29–91)	21 (88%)
HOS-ADL	81 (21, 14–100)	24 (12%)	91 (9, 56–100)	–	51 (17, 14–78)	–	–	24 (100%)
HOS-Sport	71 (26, 11–100)	36 (18%)	83 (16, 28–100)	7 (5%)	36 (18, 11–81)	6 (13%)	38 (38, 38–38)	23 (96%)
WHOQOL physical	70 (20, 7–100)	26 (13%)	77 (15, 7–100)	3 (2%)	48 (17, 14–79)	1 (2%)	50 (25, 32–68)	22 (92%)
WHOQOL psychological	76 (14, 29–100)	26 (13%)	79 (13, 29–100)	3 (2%)	68 (13, 38–92)	1 (2%)	52 (9, 46–58)	22 (92%)
Components of composite outcome								
Increase in HOS-ADL > 23	85 (47%)	24 (12%)	85 (64%)	–	0 (0%)	–	–	24 (100%)
Postoperative HOS-ADL > 80	120 (67%)	24 (12%)	120 (90%)	–	0 (0%)	–	–	24 (100%)

^a^Successful outcome is defined as an HOSADL > 80 or HOSADL increase > 23 1 year postoperative

Unless otherwise indicated values between parentheses are (sd, range)

Logistic regression was used to evaluate the association between each prognostic factor and outcome. Potential prognostic variables were entered into a logistic regression model, taking into account the multiple imputed datasets. The univariable odds ratios of the variables were calculated using univariable logistic regression analyses to evaluate their individual contribution. Multivariable logistic regression with a backward stepwise selection procedure was used to achieve the most informative and parsimonious combination of predictors. Akaike’s information criterion (*p* < 0.157) was used as a selection criterion [28, 29]. The probability of having an successful functional outcome can be calculated by using the following formula: ${\mathrm{P}}_{\mathrm{successful}\ \mathrm{outcome}=}{\mathrm{e}}^{\left({\upbeta}_0+{\upbeta}_1\ast {\mathrm{x}}_1+{\upbeta}_2\ast {\mathrm{x}}_2+\dots +{\upbeta}_{\mathrm{n}}\ast {\mathrm{x}}_{\mathrm{n}}\right)}/1+{\mathrm{e}}^{\left({\upbeta}_0+{\upbeta}_1\ast {\mathrm{x}}_1+{\upbeta}_2\ast {\mathrm{x}}_2+\dots +{\upbeta}_{\mathrm{n}}\ast {\mathrm{x}}_{\mathrm{n}}\right)}$.

In this formula, P_successful outcome_ is the probability of having a successful functional outcome, β_0_ represents the constant and β_1_, β_2_ and β_n_ are the regression coefficients of the predictors x_1_, x_2_, and x_n_, respectively, after having been pooled.

The model performance was assessed on calibration with the Hosmer-Lemeshow goodness-of-fit test and a calibration plot to estimate its reliability [29, 30]. The model’s ability to discriminate between patients with successful or unsuccessful outcomes was estimated as the area under the curve (AUC) of the receiver operating characteristic (ROC) curve of the model [31]. Prediction models derived with multivariable regression analyses are known for over fitting, which results in too extreme predictions when applied in new cases. Therefore, the model was validated internally using bootstrapping techniques. Five hundred samples were drawn with replacement from the development sample. Bootstrapping techniques provide information on the performance of the model in comparable datasets and generate a shrinkage factor to adjust the regression coefficients [31, 32]. After this adjustment, the model performance was reevaluated. A nomogram was created to easily calculate the risk of a successful outcome after hip arthroscopy for a given patient.

All statistical analyses were performed using SPSS version 22 (IBM, New Jersey, US) and R version 3.3.1 (R Foundation, Vienna, Austria) with package ‘rms’ [33].

## Results

**Fig. 1:**
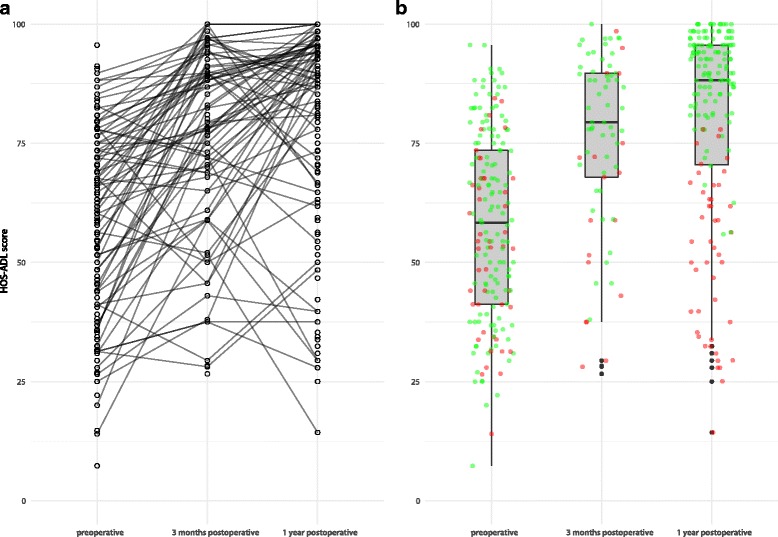
**a** Graphical representation of temporal changes in HOS-ADL scores for each patient. Please note that due to missing values for some patients scores at certain time points are not available and connecting lines not drawn. **b** Boxplots showing distribution of preoperative HOS-ADL scores and scores at 3 months and 1 year postoperative. Green dots represent patients with successful outcome after hip arthroscopy at 1 year follow-up. Red dot represent patient without successfull outcome

Our database yielded 205 patients, 203 were eligible for statistical analysis. Two patients were excluded: one because of a different indication (free body), and the other because at the start of surgery it was not possible to reach the joint space as the capsule was too tight. The latter patient was afterwards referred for a Ganz osteotomy. Out of the 203 participating patients 74% had a successful outcome 1 year after hip arthroscopy according to our composite outcome. Temporal changes (pre-operative, 3 months and 1 year) in HOS-ADL scores are shown in Fig. 1. Additional information on the two components of our composite outcome (i.e. > 23 points improvement in preoperative HOS-ADL at 1 year postoperative, or an HOS-ADL > 80 at 1 year postoperative) is presented in Table 1.

Of the 203 eligible patients 114 (56%) were female. The patients had a mean age of 40 years (SD±11), a mean BMI of 26 (SD±4), and the mean time of complaints prior to surgery was 4 years (SD±4). The indications for surgery were cam morphology (121 (60%)), pincer morphology (46 (22%)) (both causing FAI) and labral tear (138 (67%)). Patients had a mean alpha angle of 65° (SD±14). Table 1 presents the demographic and clinical characteristics of the study population.

Gender, cam, preoperative PRO scores of the HOS-ADL and WHOQOL psychological showed to be significantly (*p* < 0.05) associated with outcome in univariable analysis (Table 2).

**Table 2:** Logistic regression analysis of predictor variables for successful outcome 1 year after hip arthroscopy

Predictors		Univariable analysis		Multivariable analysis		
		Odds ratio (95% CI)	*P* value	Odds ratio (95% CI)	Regression coefficient	*P* value
Constant					–0.19	
Gender	female vs male	0.38 (0.19–0.76)	0.01	0.37 (0.17–0.83)	–0.97	0.02
Age (IQR 31–48)		1.07 (0.67–1.72)	0.77	–	–	–
BMI (IQR 23–28)		0.75 (0.48–1.16)	0.20	–	–	–
Years of complaints (IQR 2–4)		0.95 (0.81–1.13)	0.59	–	–	–
Tönnis classification	grade ≥ 1 vs grade 0	1.30 (0.36–4.66)	0.69	–	–	–
Alpha angle (IQR 52–75)		1.66 (0.97–2.83)	0.06	–	–	–
CAM	yes vs no	2.38 (1.25–4.55)	0.01	–	–	–
Pincer	yes vs no	0.58 (0.28–1.21)	0.15	0.47 (0.21–1.09)	–0.74	0.08
Labral tear	yes vs no	0.55 (0.27–1.15)	0.11	0.46 (0.20–1.06)	–0.77	0.07
Preoperative VASpain (IQR 5–8)		0.91 (0.50–1.63)	0.75	–	–	–
Preoperative mHHS (IQR 48–64)		1.44 (0.95–2.18)	0.09	–	–	–
Preoperative HOS-ADL (IQR 41–74)		1.84 (1.08–3.15)	0.02	2.01 (0.99–4.08)	0.02	0.05
Preoperative HOS-Sport (IQR 48–64)		1.38 (0.83–2.29)	0.21	–	–	–
Preoperative WHOQOL physical (IQR 48–64)		1.35 (0.88–2.06)	0.16	0.43 (0.22–0.87)	–0.04	0.02
Preoperative WHOQOL psychological (IQR 60–79)		1.69 (1.12–2.57)	0.01	2.40 (1.38–4.18)	0.05	0.002

*IQR* interquartile range. Odds ratios for continuous predictors are presented as IQR odds ratios

After backward selection, the following variables remained in the multivariable model: gender, pincer, labral tear, HOS-ADL, WHOQOL physical, and WHOQOL psychological (Table 2). The reduced model’s AUC of the ROC curve was 0.72 (95% CI: 0.65–0.80) and the Hosmer-Lemeshow goodness-of-fit test was not statistically significant, indicating that the model fits the data well.

**Fig. 2:**
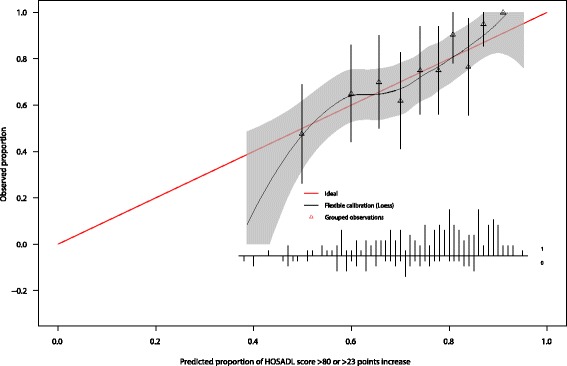
Calibration plot. Distribution of predicted probabilities shown separately for patients with and without a successful outcome after hip arthroscopy. Triangles indicate observed proportions of successful outcome after hip arthroscopy, by tenths of predicted probability

Through bootstrapping the maximum absolute difference in predicted and calibrated probabilities (E_max_) and a shrinkage factor were determined, 0.15 and 0.61, respectively. After multiplying the regression coefficients with the shrinkage factor the models performance was reevaluated. The mean probability of having a successful functional outcome was 67% (SD±12%). Female patients in our population had a lower chance on a successful outcome 1 year after hip arthroplasty compared to men (OR: 0.37 (95% CI 0.17–0.83); *p* = 0.02). Patients with indications pincer morphology (OR: 0.47 (95% CI 0.21–1.09); *p* = 0.08) or labral tear (OR: 0.46 (95% CI 0.20–1.06); *p* = 0.07) had a lower chance on a successful outcome. Patients with a higher preoperative HOS-ADL had a higher chance on a successful outcome (IQR OR: 2.01 (95% CI 0.99–4.08); *p* = 0.05). A lower score on the WHOQOL physical domain (IQR OR: 0.43 (95% CI 0.22–0.87); *p* = 0.02) and a higher psychological score (IQR OR: 2.40 (95% CI 1.38–4.18); *p* = < 0.01) gave a higher chance on a successful outcome. The final model’s discrimination yielded an AUC of the ROC curve of 0.71 (95% CI: 0.64–0.80). The model’s calibration was visualized with a calibration plot (Fig. 2). The Hosmer-Lemeshow goodness-of-fit test was not significant (*p* = 0.48), indicating that the model fits the data well.

The risk of a successful functional outcome after hip arthroscopy for a given patient can be calculated as follows:

$$ {\mathrm{P}}_{\mathrm{successful}\ \mathrm{outcome}}={\mathrm{e}}^{\left(\mathrm{lp}\right)}/1+{\mathrm{e}}^{\left(\mathrm{lp}\right)}, $$

where

$$ \mathrm{lp}=-0.19+\left(-{0.97}^{\ast }\ \mathrm{female}\right)\kern0.5em +\left(-{0.74}^{\ast }\ \mathrm{pincer}\right)+\left(-{0.77}^{\ast }\ \mathrm{labral}\ \mathrm{tear}\right)+\left({0.02}^{\ast }\ \mathrm{HOS}-\mathrm{ADL}\ \mathrm{score}\right)+\left(-{0.04}^{\ast }\ \mathrm{WHOQOL}\ \mathrm{physical}\ \mathrm{score}\right)+\left({0.05}^{\ast }\ \mathrm{WHOQOL}\ \mathrm{psychological}\ \mathrm{score}\right) $$

**Fig. 3:**
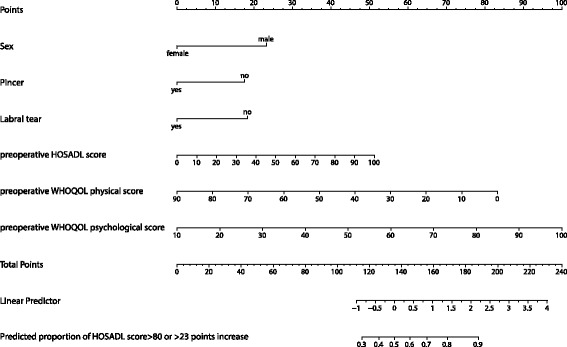
Nomogram for prediction of a successful outcome after hip arthroscopy in a given patient. To calculate the probability of a successful outcome, first obtain the value for each predictor by drawing a vertical line straight upward from that predictor to the points’ axis, then sum the points obtained for each predictor, and locate this sum on the total points’ axis of the nomogram, where the probability of a successful outcome after hip arthroscopy can be located by drawing a vertical line downward

The nomogram created as a tool to easily calculate the risk of a successful outcome after hip arthroscopy for a given patient is shown in Fig. 3.

## Discussion

Arthroscopic procedures for FAI caused by cam/pincer morphologies or labral tears, can significantly resolve complaints and impairment in patients. However, not all patients equally benefit from this procedure. Careful patient selection is of great importance for success of this procedure. In this study a clinical prediction model was developed using logistic regression for functional outcome 1 year after hip arthroscopy, containing six easy accessible preoperative risk factors: gender, indication: pincer and labral tear, and the preoperative PRO scores: HOS-ADL and WHOQOL physical and psychological domains. Based on this model, a nomogram was created that can be used to easily calculate the risk of a successful outcome after hip arthroscopy for a given patient.

The discriminating accuracy of this model as assessed by the AUC of the ROC curve turned out to be fair, 71% (95% CI: 64–80%) of the patients were classified correctly. The model has a relatively high predictive probability (67%) for successful outcome after hip arthroscopy, as most patients in the cohort had a successful outcome after surgery. The developed model is a first step to predict the course of functional outcome of patients that are considered for a hip arthroscopic intervention, as its accuracy can still be improved through external validation to examine the generalizability for other hip arthroscopic populations [30]. However, the model can be used as a guidance tool to optimize preoperative decision-making.

Female gender was identified as a predictor in the final model. Findings out of previous studies on gender as a predictor showed to be inconsistent [34, 35]. The study of McCarthy et al. [34] identified predictors for long term survivorship after hip arthroscopy and analyzed that gender had no predictive value. The study of Frank et al. [35] compared clinical outcomes (HOS and mHHS) before and after hip arthroscopy and pointed out that gender was predictive for both HOS-Sport and mHHS. Women presenting with hip pain have different hip morphology compared to men (smaller alpha angles, increased acetabular version, and increased femoral anteversion), and speculations are made that this difference is caused by a greater component of soft-tissue laxity and difference in muscle mass, as it results in less protective dynamic stabilization of the painful hip joint [36]. Also hip dysplasia is known to have a higher occurrence in women and can lead to inferior results and higher failure rates after arthroscopic treatment of FAI [16].

Our model identified pincer morphologies and labral tear indications as predictors that have a negative effect on a successful outcome. Multiple studies demonstrate that cam and pincer morphologies and labral tears induced FAI, in the absence of significant degenerative changes, are appropriate indications for arthroscopic hip surgery resulting in improvements in functional outcome [37, 38]. In our population patients with these indications had a lower chance to get the desired improvement in functional outcome. Therefore, more cautious consideration is advised compared to patients with cam impingement.

The other predictors in our model were based on preoperative PRO scores. Preoperative HOS-ADL showed to be a predictor in the prediction model. That a preoperative outcome score can have predictive value in predicting postoperative outcome seems logical, but there is still limited evidence on this subject, as only Philippon et al. [11] identified the preoperative mHHS as predictor for postoperative outcome. The physical and psychological domains of quality of life, based on the WHOQOL-BREF, were also identified as predictors in the final model. There have been no studies known by the authors to use this quality of life score as a predictor for functional outcome. Yet, there are studies that describe a strong correlation between psychological factors and post-operative outcome in other fields of orthopedic surgery (including total joint arthroplasty, anterior cruciate ligament reconstruction, and spine surgery for degenerative disease) [39]. Several intervention strategies exist to address these psychological factors when they appear to contribute suboptimal postoperative rehabilitation or recovery [39].

Previous studies show there is evidence that demographic factors such as age, BMI and duration of symptoms can be predictors of outcome after hip arthroscopy, but are inconsistent [7, 9–11, 13–15, 34, 35]. Our model does not show this predictive relationship either. However, some of the risk factors (the cam type FAI (IQR OR: 2.38 (95% CI 1.25–4.55); *p* = 0.01) and preoperative alpha angle (IQR OR: 1.66 (95% CI 0.97–2.83); *p* = 0.06)) that did not make it into the final predictive model, showed to have a correlation with successful outcome in univariable analysis.

In addition to assisting clinicians in patient selection for hip arthroscopic interventions, the model can be used for consulting patients on their expectations of successful surgery. A study examining satisfaction in total knee arthroplasty patients found that preoperative expectations affect satisfaction [19]. As patients with a lower risk score have a lower chance on a successful outcome, patients and clinicians should adjust their expectations accordingly.

Some potential limitations of our study have to be discussed. We had to define improved functional outcome after hip arthroscopy (composite of HOS-ADL score above 80 or increase of 23 points). Despite the limitations related to the use of composite outcomes, the impossibility for patients with preoperative HOS-ADL scores of > 80 points to increase 20 points or more necessitated the use of a composite outcome (as these patients otherwise would have been considered unsuccessful irrespective of their score at 1 year postoperative). The HOS-ADL was chosen as the main outcome score because it is a validated, self-administered score and is designed for younger patients with hip pathology without relevant arthritic degeneration [40–42]. The cutoff value for improved outcome was based on the MCID of 23 determined in a recent study done by Chahal et al. [25] Other studies show different MCID values, e.g. Martin et al. [43] found an MCID of 9 (which we considered to be too low to be of clinical importance). Repeating the analysis with a cutoff based on this MCID (HOS-ADL score above 80 or increase of 9 points) resulted in a very similar model, yielding the same predictive factors as our current prediction model. Furthermore, in order to use this prediction model, the suggested PROs have to be used.

There are also limitations in our follow-up duration, population size and missing values in the outcome scores. Our study has a relatively short follow-up time (1 year) and a small study group size (205), although it is larger than presented by most previous authors. Models developed from datasets with too few outcome events relative to the number of candidate predictors are likely to yield biased estimates of regression coefficients. They lead to unstable prediction models that are overfit to the development sample and perform poorly on new data. It has been suggested that an EPV of 10 or more is needed to avoid the problem of overfitting [44–46]. To make sure that the model would not overfit the data, the number of variables included in the model was kept within the limit of 10 events per predictive variable. Another limitation is the influence of pre-, peri- and postoperative factors on functional outcome. All patients had a standardized preoperative selection process, based on known indications, contraindications and the surgeon’s clinical expertise. Outcome can also be affected by factors as perioperative findings or treatment, complications during surgery or injuries after surgery. Examples are, e.g. unexpected chondral damage based on the Outerbridge classification [7, 10, 11, 13, 34], labral repair versus debridement [11], and residual FAI after surgery [47]. These factors are not in the prediction model but still influence the outcome of hip arthroscopic interventions.

Finally, this study included a relatively diverse range of preoperative risk factors, as the addition of preoperative PRO scores as potential risk factors, which is unique in this research field. However, not all possible preoperative risk factors were included in the study. For example, risk factors based on physical examination [48] or radiographic measurements (CT/MRI) [9, 49] can still be added to the model, as every predictor adds to a more accurate identification of patients at risk for an successful outcome.

## Conclusion

This study identified six easy accessible preoperative risk factors that can be used to predict functional outcome 1 year after a hip arthroscopic intervention, i.e. gender (female), indication (pincer and labral tear), HOS-ADL (low), WHOQOL physical (high) and WHOQOL psychological (low) score. The proposed clinical prediction model is a first step to predict the functional outcome of patients that are considered for a hip arthroscopic intervention, as it can still be improved through external validation and/or adding additional potential predictors.

## Abbreviations

95% CI: 95% Confidence interval
ADL: Activities of daily living
AUC: Area under the curve
BMI: Body mass index
CT: Computed tomography
EPV: Events per variable
FAI: Femoroacetabular impingement
HOS: Hip outcome score
IQR: Inter-quartile range
MAR: Missing at random
MCID: Minimal clinically important difference
mHHS: Modified Harris Hip score
MRI: Magnetic resonance imaging
OR: Odds ratio
PRO: Patient reported outcome
ROC: Receiver operating characteristic
THA: Total hip arthroplasty
TRIPOD: Transparent reporting of a multivariable prediction model for individual prognosis or diagnosis
VAS: Visual analogue scale
WHOQOL-BREF: World Health Organization’s quality of life assessment (abbreviated version)
